# Cardiovascular Tissue Engineering Models for Atherosclerosis Treatment Development

**DOI:** 10.3390/bioengineering10121373

**Published:** 2023-11-29

**Authors:** Linnea Tscheuschner, Abraham R. Tzafriri

**Affiliations:** 1 Department of Vascular Surgery, National and Kapodistrian University of Athens, 15772 Athens, Greece; 2 Department of Research and Innovation, CBSET Inc., Lexington, MA 02421, USA; rtzafriri@cbset.org

**Keywords:** tissue engineering, disease models, atherosclerosis, nanoparticles, drug-eluting stents, drug-coated balloons

## Abstract

In the early years of tissue engineering, scientists focused on the generation of healthy-like tissues and organs to replace diseased tissue areas with the aim of filling the gap between organ demands and actual organ donations. Over time, the realization has set in that there is an additional large unmet need for suitable disease models to study their progression and to test and refine different treatment approaches. Increasingly, researchers have turned to tissue engineering to address this need for controllable translational disease models. We review existing and potential uses of tissue-engineered disease models in cardiovascular research and suggest guidelines for generating adequate disease models, aimed both at studying disease progression mechanisms and supporting the development of dedicated drug-delivery therapies. This involves the discussion of different requirements for disease models to test drugs, nanoparticles, and drug-eluting devices. In addition to realistic cellular composition, the different mechanical and structural properties that are needed to simulate pathological reality are addressed.

## 1. Introduction

Tissue engineering was originally defined as an interdisciplinary field, aimed at generating biological tissue to replace, maintain, or improve natural tissue function [1].

Since the dramatic demonstration by Vacanti’s group showing the ability to grow an ear-shaped cartilage structure under the skin of a mouse [2], tissue engineering has been focused on generating healthy tissue or organs to fill the increasing gap between organ demand and actual donations. Despite this hyperfocus, it took about 20 years until the first human-size organs like a heart could be generated [3] and even to date actual clinical human trials with tissue-engineered organs are sparse [4]. As the field evolved, other opportunities and applications came into focus. In particular, due to the lack of complexity in simple 2D cell cultures and suitable animal models, tissue engineering expanded to a discipline also aiming to generate suitable disease models in various biomedical research fields [5,6].

Generating functional vascular tissue was the main goal in cardiovascular tissue engineering in the last decades [7] in order to replace tissue affected by cardiovascular disease (CVD).

The term “cardiovascular disease” does not refer to a single disease, but to a cluster of different impairments of the cardiovascular system. This includes coronary heart disease (CAD-disease affecting the vessels supplying the heart muscle), cerebrovascular disease (disease affecting the vessels supplying the brain), and peripheral arterial disease (PAD-disease affecting the vessels supplying arms and legs) [8].

**Figure 1:**
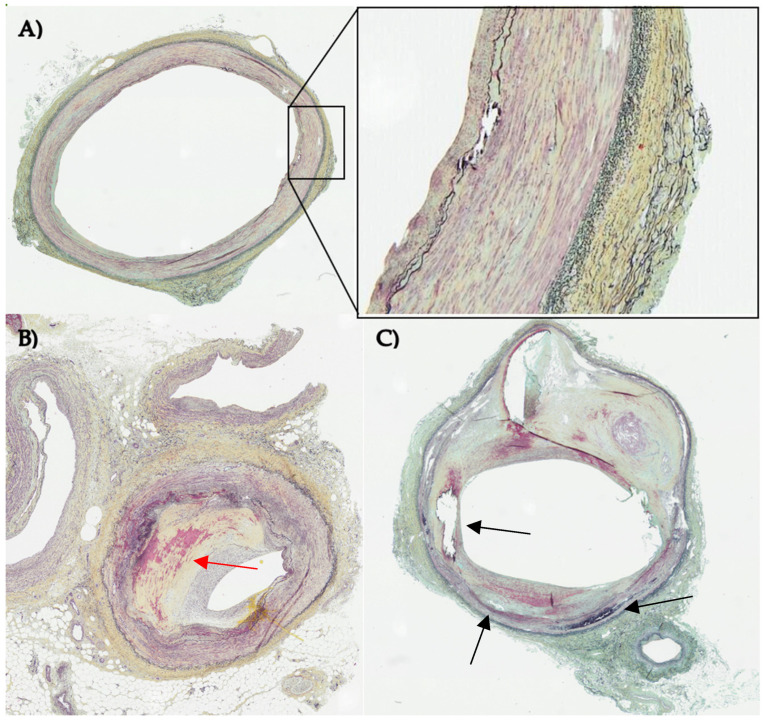
Histological sections of three different human lower limb arteries stained with Movat pentachrome staining. Nuclei are colored in black, fibrin and muscle in red, collagen in yellow and elastic fibers in blue/black. (**A**) vessel in early disease state with an organized layered structure (close up) and slightly visible intimal hyperplasia (**B**) atherosclerotic vessel with severe stenosis due to intimal hyperplasia (red arrow) (**C**) diseased vessel with disrupted vascular structure with calcification (black arrows) and lipid core. Images were allocated by CBSET Inc., Lexington, MA, USA (CBSET = Concord Biomedical Engineering and Emerging Technologies incorporated).

For all those diseases, the underlying cause is arterial disease, predominantly atherosclerosis. The healthy tri-layer ultrastructure, comprising of an inner intima, media, and outer adventitia, is disrupted by cellular infiltrates and the deposition of fat, fibrin, and minerals to form so-called plaques or lesions [9,10,11] (Figure 1).

Plaque progression is characterized by the degree of luminal stenosis of the vessel [11]. Until the lesion leads to a significant stenosis that limits blood supply in the downstream tissue, it remains clinically silent. But studies show that plaques can progress very fast and that thin-cap atheromas (vulnerable plaques) tend to rupture, which can lead to acute events like stroke or heart attack [11].

Delayed diagnosis and lack of treatment options that go beyond risk minimization and inhibition of disease progression, result in an estimated death each 34 s due to CVD only within the United States [12].

As early as 1986, the first tissue-engineered vascular grafts (TEVG) emerged as a substitute to these diseased vascular areas [13]. Already extensively reviewed by others [14,15,16], the field of tissue-engineered vascular structures has advanced over the past decades and TEVG have been successfully implanted into patients in various clinical trials (Table 1). But these artificial vessels, especially those with a small diameter, show major complications, like risk of thrombosis and lack of patency [17].

Especially, the need for a suitable matrix for a tissue-engineered blood vessel (TEBV) with physiological burst pressures, compliance, and mechanical properties limits the generation of implantable constructs [15]. To overcome this issue, many sophisticated approaches were undertaken in recent years. An initial approach is to use porcine tissue to generate scaffolds, by decellularized pericardium or arteries with the desired diameter directly [27,28,29]. These decellularized scaffolds maintain physiological mechanical properties [29] and can be recellularized with porcine vascular cells [28] or human-derived cells [27], presenting TEBV with histological and mechanical characteristics comparable to human vasculature.

A non-xenograft approach for generating vascular matrix for tissue engineering of small-diameter blood vessels is the use of human umbilical arteries (hUA) [30]. After decellularization of hUA [31], the cell-free matrix shows sufficient structural protein and biomechanical properties preservation [30] and can be repopulated, for instance, with mesenchymal stem cells [32] or endothelial cells, resulting in TEBV with biological and mechanical properties that withstand in vivo implantation [33].

Other synthetic approaches utilize electrospinning to generate scaffolds for TEBV construction [27]. Atala and his group used a bilayer approach to fabricate a scaffold with optimized pore sizes in each layer for enhanced attachment of endothelial cells in the inner layer and enhanced attachment of smooth muscle cells in the outer layer of the construct [34]. To combat the burden of thrombogenicity of small-vascular grafts, they developed a novel approach of combining electrospinning for scaffold generation wrapped with a layer of a smooth muscle cell sheet fabricated with a temperature-responsive polymer [35], leading to biological matured and biomechanical functional construct, even under physiological flow conditions.

The ability to generate functional tissue with 3D bioprinting made it feasible to print vascularized tissue or tissue-engineered vascular grafts [36]. After optimizing material selection and printing conditions, 3D bioprinting enables the generation of complex TEBV with a high throughput [37].

Recently, 4D printing is emerging as an advancement of 3D printing, wherein the printed materials are designed to change their properties, like size, shape, or function, over time, triggered by an external stimulus [38]. These stimuli-responsive materials are considered as “smart materials” and find increasing application in the field of bioprinting [39,40,41,42,43].

Smart materials that show shape-sensitive behavior to a temperature stimulus have been used for generating polymeric vascular grafts [44] and were successfully tested in vivo in a mouse model [45]. The TEVG was designed to be compact at low temperature, while expanding to its target size in response to ambient heating. To achieve this, the blood flow was temporally restricted prior to the implantation, and the compact form of the graft was inserted into the animal’s aorta. After successful positioning, blood flow was restored and a rise above the threshold temperature triggered an expansion of the TEVG to its final conformation [45].

These smart materials can also be seeded with cells and have been implanted in vivo as patches to treat cardiac infarction [46]. Additionally, it was shown that vascular tubes consisting of ion-responsive hydrogels and murine bone marrow stromal cells could be generated from a 4D bioprinting approach, leading to vessel structures with a small diameter of 20 µm [47]. Creating these small diameter vessels to mimic the capillary vasculature structure to perfuse bioprinted tissue-engineered organs remains a major limitation in bioprinting of functional tissues [48]. Other novel approaches employ external biological stimuli to trigger self-assembly of different bioprinted cellular spheroids as “bio bricks” to mimic multilayer arteries [49].

With the increasing complexity in material selection and optimization of printing processes the emergence of artificial intelligence (AI) based on machine learning or deep learning algorithms can support the fabrication of 4D bioprinted constructs [50,51]. Indeed, AI is finding use in all the key design and fabrication steps, from medical imaging reconstruction to generating patient-specific geometries for 3D/4D bioprint blueprints [52], through the selection of printing materials, optimization of the implementation process, and the selection of stimulus conditions [53].

Mirroring a broad trend in tissue engineering, vascular tissue engineering underwent an important evolution from solely focusing on generating healthy tissue substitutes to increasingly focusing on generating and replicating diseased tissues for research and treatment development purposes.

Particularly, the study of onset and initial progression of cardiovascular disease and the testing of clinical treatments creates a strong demand for tissue-engineered disease models, a need that 2D cells cultures and animal models cannot fill.

This review is aimed to serve as a guideline for the generation of adequate disease tissue engineering models for testing and refinement of treatment strategies for cardiovascular disease. For this purpose, we analyze existing tissue-engineered models of atherogenesis and advanced atherosclerosis and evaluate their potential use for treatment testing according to selected complexity criteria. The relevance criteria were chosen to reflect the necessary attributes for systemic drug treatment, nanoparticles, and endovascular drug-delivery devices.

Here we distinguish between models that are created to study atherogenesis and the progression of the disease in an early state, and those that focus on advanced disease state. Models of early disease states aim to reflect the cellular, structural and mechanical properties of healthy vessel environment as the backdrop for the emergence of pathologies. In addition, we focus on models that aim to depict advanced stages of the disease, which could be used to test and optimize different treatments of the disease in a clinically relevant stage.

Creating advanced disease is more challenging, since the physiological environment of these lesions is rather complex. Instead of only native tissue cells arranged in an organized structure, advanced diseased states show migration of immune cells and severe disorganization in the structure of the vascular wall [54]. Additionally, various non-cellular components accumulate within the plaque, leading to stenosis of the affected vessel. Depending on the classification of the lesion, the core can contain a necrotic core, large lipid pools (lipid plaques), can be covered with a fibrous cap (fibrotic plaque) or show severe calcification (calcified plaque) [54] (Table 2).

Despite its globally high prevalence, the treatment options for atherosclerosis disease are very limited. The primary approach is enforcing a lifestyle change to minimize disease progression [55].

The medical treatment of atherosclerosis is mainly limited to agents that either reduce disease risk factors, like lowering LDL (lower density-lipoprotein) levels (e.g., Statins) or hypertension (e.g., Angiotensin-converting enzyme Inhibitors) [56], or minimize the risk of complication and thrombosis by applying e.g., anticoagulants or antiplatelet medication [57]. More recently approaches are taken to target the progression of the disease directly by inhibiting the inflammatory reaction within the lesion [58].

A novel attempt is the utilization of nanoparticles in the diagnosis and treatment of CVD. On the one hand, nanoparticles are used to enhance the sensitivity of lesion imaging with nanoprobes, improving disease detection at an early state [59,60,61,62]. On the other hand, nanoparticles are used as drug-carriers that target the inflamed lesion tissue and inhibit the progressing inflammatory process [63,64,65].

Generally, for the treatment of advanced CAD and PAD, endovascular approaches and surgical procedures build the foundation of treatment. Often these procedures are complementary and applied in a hybrid manner [66]. To select the most effective treatment approach parameters like location, morphology and lesion complexity should be taken into consideration [67]. Mostly endovascular treatments are considered as primary treatments in patients with critical limb ischemia [68]. Endovascular procedures include stenting, drug-eluting stenting, plain balloon angioplasty, drug-eluting balloon angioplasty, atherectomy or a combination of multiple procedures.

Traditional models to study cardiovascular disease and potential treatments are either simple 2D cell cultures or complex animal models. In 2D cell cultures, one or multiple cell types (co-culture) are cultivated in a monolayer attached to a surface of a petri dish or culture flask [69]. These cultures are extensively used for fundamental research purposes or high throughput drug screening, because of their simple and low-cost maintenance. It is apparent that these very simplified 2D models have several limitations and are not able to depict the complexity of a diseased tissue on multiple levels [70].

Newer approaches, use “organ-on-chip” microfluidic devices to connect different monolayered cell cultures to analyze disease behavior and drug efficiency in a multi-cellular system [71]. Being designed to be perfused by flow, this technique is primed to be utilized for cardiovascular mimicking. But unless, the “organ” on the chip is a suitable vessel construct rather than a single cell monolayer, these devices lack complexity for vascular modeling.

In contrast, animal models can serve a considerably higher level of complexity and are adequate to study a range interaction based on biological, chemical or physical factors.

Over the course of the last decades many sophisticated animal models have been developed for atherosclerosis research [72,73,74]. Most commonly apolipoprotein E (ApoE) knock-out mice are used to study atherogenesis and progression, since these mice are able to express the gamut of clinically relevant advanced lesions [75]. On the other hand, porcine models are commonly used to test interventional cardiovascular procedures and treatment approaches, due to their similar vascular anatomy, electrophysiological signal and flow conditions [76]. Pigs can develop spontaneous atherosclerotic lesions, which can be accelerated by an atherogenic diet [72]. This process can be even further accelerated by introducing mutations targeting the lipid metabolism like the LDL receptor or knockouts of the ApoE protein [77,78]. Nevertheless, these animal models contain limitations in holistically emulating the human vascular anatomy, disease progression or biochemical pathways. Depending on animal species and size, reproduction and maintenance can be labor-intensive, expensive and take time and space. Above all, animal experiments are always accompanied by ethical considerations and regulatory requirements [79,80].

3D tissue-engineered disease models could facilitate the ideal completion between these models, being low-maintenance, high throughput and able to simulate diverse tunable variables.

**Figure 2:**
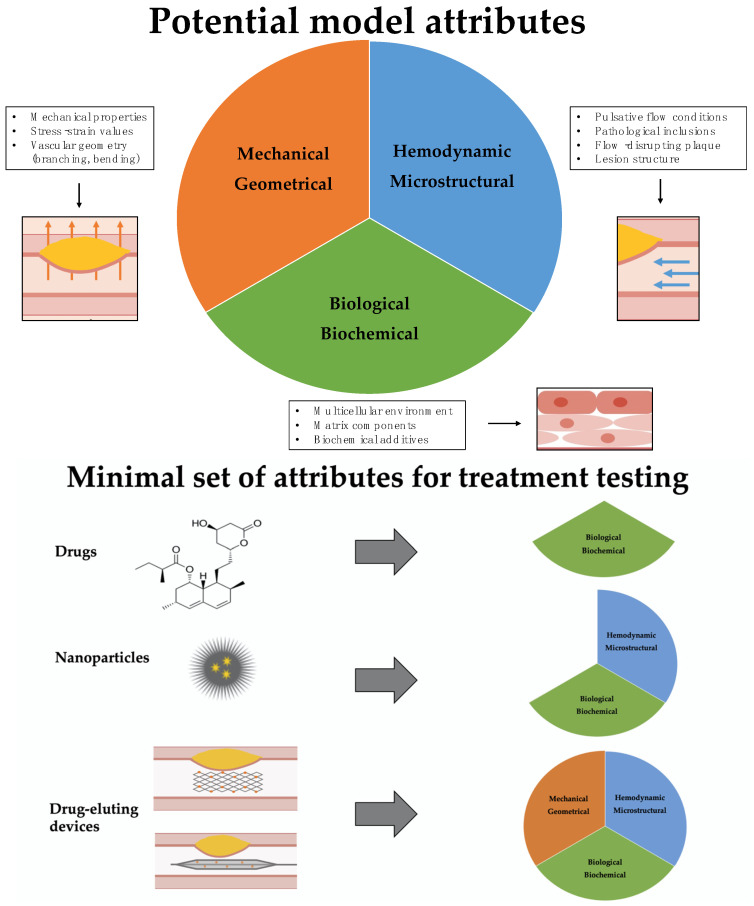
Upper part: Overview of key model attributes. In this review we evaluate existing tissue engineering models according to their ability to mimic healthy and diseased vascular tissue focusing on three attribute categories: biological–biochemical environment, hemodynamic–microstructural conditions (blue arrows), and mechanical–geometric (orange arrows) properties. Lower part: Overview of the minimal necessary set of attributes that a model has to represent for testing three different treatment approaches. While testing of drugs can be performed on models that replicate the biological–biochemical environment of the tissue, therapeutics that are based on nanoparticles need to also include a hemodynamic-microstructural aspect of the vascular environment, since the delivery and attachment of the particles is influenced by blood flow. Testing of therapies based on drug–eluting endovascular devices requires models that holistically replicate the biological–biochemical, hemodynamic–microstructural and mechanical–geometrical structure of the vascular system, since drug delivery is also dependent on device tracking and mechanical inflation.

In this review we classify the tissue attributes that need to be modelled by the tissue-engineered construct in three different complimentary classes: biological–biochemical, hemodynamic–microstructural, mechanical–geometrical (Figure 2). The biological–biochemical category refers to the (multi-)cellular environment of the model, that is embedded in a biochemical matrix. Also, the ability to add different biochemical components like cytokines or oxLDL is included. With the hemodynamic–microstructural category, we are referring to the ability to apply physiological flow and flow–disrupting components like the plaque itself. The vascular structure around the lesion and the structure of the lesion itself is defined as “microstructure” here. Under the category of “mechanical–geometrical” attributes falls the mechanical properties of the lesion and the surrounding tissue, as well as the more global vascular structure surrounding the lesion that might contain bending or branching (Figure 2). The relevance of each attribute class varies with the treatment approach, with the minimal set of required attributes increasing from drug screening to testing of nanoparticles and endovascular drug-delivery devices (Table 3 and Figure 2).

## 2. Material and Methods

A PubMed search was performed under the terms “disease-inspired tissue engineering“, “atherosclerosis tissue engineering“, “vascular disease tissue engineering“, “tissue-engineered blood vessel“, and “atherogenesis tissue engineering“ from the years 1970 to 2023.

From the resulting 2753 publications, only publications covering the topic of tissue-engineered models relevant for studying atherogenesis and atherosclerosis were further investigated for this review.

This was supplemented by a manual search through the bibliographies of landmark papers for further relevant articles. The resulting pool of publications about atherosclerotic tissue engineering models were analyzed for including the three here-defined attributes “biological–biochemical”, “mechanical–geometrical” and “hemodynamic–microstructural”.

Of these, 17 publications that contained relevant models were selected to be discussed in greater detail.

## 3. Tissue Engineering for Systemic Drug Treatment

Drugs targeting the risk factors for CVD, e.g., statins or beta-blockers, have been the most commonly prescribed drugs in the US for years [81]. Most of these drugs are circulating systemically and target cells in specific organs like liver or heart cells and modulate, for example, the blood pressure or cholesterol levels.

**Figure 3:**
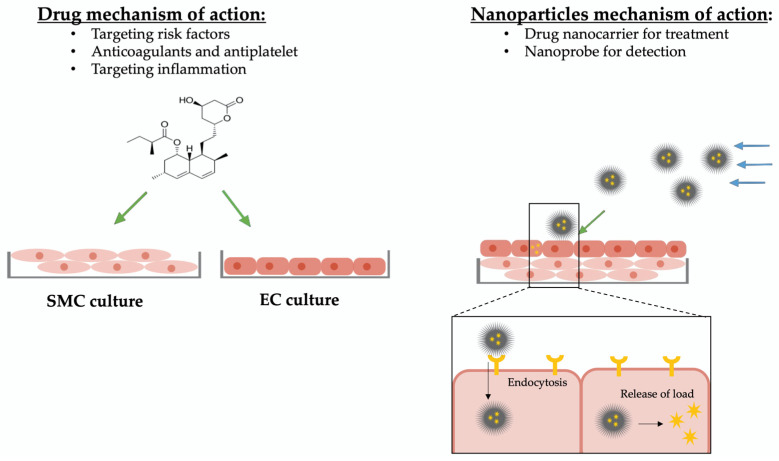
Overview mechanism of action of drugs and nanoparticles. Drugs targeting atherosclerosis are designed to have a non-selective effect on specific cell types and are screened on simple cell cultures. For studying and testing of new drugs, mimicking the biological–biochemical tissue environment is sufficient. Nanoparticles circulate in the blood flow, approach their specific target tissue selectively, and release their molecule of interest (drug or nanoprobe). To study and optimize nanoparticles of atherosclerosis treatment, not only the biological–biochemical environment but also the hemodynamic–microstructural conditions need to be simulated. Impact due to biological–biochemical attributes are indicated with green arrows, impact due to hemodynamic–microstructural conditions are indicated with blue arrows.

Novel approaches aim to target the inflamed vascular tissue directly and inhibit the progression by downregulating the inflammatory reaction [82] (Figure 3).

### 3.1. Model Attributes

In order to discover and screen for new drug approaches, simple tissue-engineered models that adequately represent the biological–biochemical environment of the tissue are used [83].

Depending on whether the model is created to screen for drugs that target atherogenesis or advanced disease, the biological–biochemical environment of a healthy or diseased vessel needs to be reproduced.

In healthy blood vessels, three different cell types that are potential targets for drug treatment can be found. The endothelia cells (EC) can be found as a monolayer in the inner layer of the artery (so-called “intima”), covered by multilayers of circular arranged smooth muscle cells (SMC) (“media”), which control the lumen diameter by contraction and relaxation [84]. The third cell type are the fibroblasts that support, together with fibro-elastic connective tissue, the vessel structure (“adventitia”) [85] and are, therefore, rarely in focus as a potential drug target.

This ordered cellular composition of the vessel is disrupted by arterial disease as a result of the migration of immune cells [86] and leads to the emergence of different drug-targets and chemical requirements for the drug properties.

Early onset of atherosclerosis is histologically characterized by the occurrence of primary intimal thickening and fatty streaks within the intima [87]. The thickening of the intima is due to increased proliferation of SMC and migration of those cells from media to intima, abolishing the separation of both cell types [88], making co-cultures useful for drug screening. As an initial inflammatory response, monocytes migrate into the intima and take up lipids (cholesterol) to form lipid-loaded macrophage, the so-called foam cells, leading to the presence of new cell types in the vascular wall [89].

With increasing progression, the inflammatory response intensifies and other immune cells, like T-cells or B-cells, migrate to the lesion [90], providing new drug targets, especially for anti-inflammatory drugs [91].

### 3.2. Available 2D and Animal Models

The primary steps in new drug discovery are high throughput methods, starting from compound screening on 384-well plates with simple 2D cell cultures, followed by secondary mechanistic assays in a more complex cellular environment [92]. Only 5–10% of the candidates make it through this process [93] and are pre-clinically tested on different animal models [94].

In vivo models provide a more complex environment to test atherosclerosis treatment and allow the assessment of drug metabolism, immune response, and other systemic effects.

### 3.3. Tissue-Engineered Models for Atherogenesis

As described above, suitable disease models for the screening and testing of drugs need to reflect the biological and biochemical environment to analyze, e.g., cytotoxicity, or study drug mechanisms.

Often these studies are performed on SMCs, ECs, or a combination of both. To meet the needs of a replenishable source of SMCs, multiple groups established protocols to differentiate functional SMCs from induced pluripotent stem cells (iPSC) with high yield [95,96,97]. This technique has also been used to generate iPSCs from individuals with genetic mutations that lead to cardiovascular diseases [98,99,100]. SMCs or ECs derived from these diseased iPSC progenitors can be used to create tissue-engineered disease models for specific genomic conditions and are useful for simple screening of new therapeutic drugs.

More advanced models aim to not only culture the vascular cells separately, but also culture different cell types in a layered structure to simulate the vascular wall composition. Su et al. [101] replicated the vascular layers on a flat chip surface by culturing an EC layer and a SMC layer separated by a subendothelial layer. This simple model could be triggered into showing early atherosclerosis phenotypes like SMC migration and EC inflammation after only 48 h by adding proinflammatory cytokines like IL-1*β* and TNF-*α* or ox-LDL to the flow cell medium. They also were able to use this early-stage disease model for drug-screening purposes.

### 3.4. Tissue-Engineered Models for Advanced Atherosclerosis

Most commercially available medications targeting cardiovascular disease are solely aiming to counteract the progression of the disease (e.g., statins) or to minimize complication in case of thrombus or plaque rupture (e.g., anticoagulants, anti-platelet agents). Recently more efforts have been undertaken to directly target the advanced lesion with anti-inflammatory and immunomodulating therapies [82].

In order to develop and screen for drugs targeting advanced stages of atherosclerosis, biological–biochemical complex tissue-engineered disease models need to be utilized.

One of the few models that aim to recreate the cellular composition of a fibroatheroma in a 3D spheroid model was published in 2018 [102]. Using a hanging drop protocol, myeloid or THP-1 derived monocytes, which were primed with pro-inflammatory markers, were co-cultured with myofibroblasts to form the so-called pseudo-plaques (ps-plaque). To form the fibrotic cap, a layer of primed human umbilical vein myofibroblasts (HUVM) were added on top of the bioengineered spheroid structure. Compared to real human plaques, large similarities could be detected in the cellular population of the ps-plaques. Even extracellular components like collagen depositions and lipid aggregates were observed.

While nicely approximating the cellular and biochemical environment of an advanced plaque, containing extracellular matrix components and local immune cells, this model lacks the involvement of blood-derived migrated immune cells.

The first real-time imaging of leucocyte recruitment was performed by a different group, using a TEBV with ECs and SMCs separated into two layers and without taking flow conditions into account. They were able to monitor increased monocyte adhesion and migration due to TNF-α expression. Vice versa, they used their TEBV model to demonstrate reduced adhesion and migration, when treated with anti-TNF-α or NF-κΒ pathway inhibitor [103].

This model could be utilized to study drugs that target the inhibition of the inflammatory reaction within advanced atherosclerotic lesions.

## 4. Tissue Engineering for Nanoparticles

In comparison to drug molecules, nanoparticles act as drug-delivery carriers with the ability to specifically target cells and deliver drug agent or labeling for detection. Regardless of being administered through inhalation, oral administration, transdermal or IV injection [104], nanoparticles that are designed to target vascular tissue eventually end up travelling through the blood stream to approach the target tissue. After reaching its target cell, the nanoparticle enters the cell and releases its load (label or drug component) [64] (Figure 3).

### 4.1. Model Attributes

To successfully test and optimize nanoparticles for the treatment of CVD, a model needs to depict the biological–biochemical environment of the diseased tissue, to test targeting efficiency, and mechanism of action. Modifications in the cellular and biochemical composition of the tissue lead to an alteration in present nanoparticle targets and distribution kinetics of drug in the tissue [105,106,107,108].

Since nanoparticles targeting CVD mostly travel through blood [109] and selectively adhere to their target cell, a suitable tissue-engineered model needs also to approximate physiological flow conditions [110]. The flow conditions determine the accumulation, retention, and wash-out of the nanoparticles [111]. Additionally, the model needs to be organized in a vascular wall-like multi-layered structure to test penetration capabilities.

Disease-originated alteration in the structural composition can lead to varying penetration and distribution behavior of nanoparticles [112]. Advanced lesions show major disorganization of vascular structure (Figure 1B,C)) and can contain additional elements, e.g., a lipid core or necrotic material. Some advanced lesions can also be covered with fibrous capsules on the luminal side (so-called “fibroatheromas” [113]), potentially changing the attachment behavior of nanoparticles and can be nanoparticle targets themselves [114].

### 4.2. Available 2D and Animal Models

The first in vitro assays for testing nanoparticles are similar to conventional drug-screening methods. The particles are tested for cell toxicity and other safety assessments on simple 2D cell cultures [115]. To further study targeting efficiency and mechanism of action, more complex models are needed. Traditionally disease-induced animal models are used to evaluated and analyze the efficiency and mechanism of nanoparticles in CVD [116]. Mostly mouse, rat, rabbit, or pig models are used for this purpose. While each model can depict one facet of the disease well, no animal model, so far, holistically captures the complexity of the disease [72].

### 4.3. Tissue-Engineered Models for Atherogenesis

As discussed above, nanoparticles designed for atherosclerosis disease approach their target lesion from the blood stream. Accordingly, suitable tissue-engineered disease models need to include key hemodynamic–microstructural attributes of the blood vessel.

For this purpose, tissue-engineered blood vessels can be used, since these often multilayered tissue constructs are arranged in a tubular shape, with enough mechanical strength for perfusion.

A simple construct was created by embedding human neonatal dermal fibroblasts (hNDF) or mesenchymal stem cells (MSC) into a rat-tail collagen I matrix and formed tissue-engineered blood vessels by plastic compression, mounted with an endothelial layer [117]. These constructs were strong enough to be perfused with physiological shear stress within only 3 h of fabrication. The TEBV showed physiological vasoactivity and vascular inflammation could be stimulated by TNF-*α* administration. Additionally, the model was used to test the effect of lovastatin on early disease state and could potentially be used for other drug and nanoparticle screenings.

Other groups used biodegradable scaffold matrixes to generate a two-layered 3D model consisting of a confluent endothelial monolayer supported by a base of multiple layers of SMCs [118]. After three days, these constructs were strong enough to be perfused with up to 10 L/min dynamic flow. To trigger initial atherogenesis and simulate early stages of atherosclerosis, the 3D model was perfused with TNF-*α* or LDL to activate the endothelium, followed by circulating monocytes, which successfully transmigrated into the intima.

As an additional approach, various groups developed vascular structures on a chip model [101,119]. Mallone et al. [119] used a computational fluid dynamic (CFD) simulation approach to first compute the most probable hotspots for LDL accumulation and verified their model on a fluidic device containing small 3D vascular grafts. They used iPSCs not only as a source for iSMCs but also to differentiate ECs. These iECs showed no significant difference in flow cytometry, or immunofluorescent and gene expression analysis to HUVEC or HBMEC cell lines. The vascular tissue engineering model was used to validate their in silico prediction model of athero-prone regions by introducing LDL and macrophage precursor (MPs) cells also derived from iPSCs (iMPs). After 4 weeks, they could observe plaque-like structures in the intima region colonized by CD11b+ macrophages and matured from the iMP source.

Being able to not only perfuse small molecules like TNF-*α* through the model, but also cells, makes these models promising candidates to be used as platforms for drug and nanoparticle screening.

To mimic an even more realistic cellular microenvironment for diseased TEBV, Zhang et al. [120] designed a 3-layer TEBV with an additional fibroblast layer around the SMC/EC layer by plastic compression. Being able to perfuse the model with physiological shear stress and addition eLDL or TNF-*α*, they could trigger early-stage atherosclerosis in this model. This was characterized by atherosclerotic hallmarks like increased monocyte accumulation and migration, foam cell formation, and general local inflammation. Additionally, the model was utilized as a drug-testing platform and they were able to demonstrate that the administration of lovastatin or NF157 lowered inflammation markers and foam cell formation. The advanced cellular and structural composition and the ability to apply physiological flow conditions make this model also fitting to test therapeutic approaches based on nanoparticles [120].

### 4.4. Tissue-Engineered Models for Advanced Atherosclerosis

As atherosclerosis progresses, the formation of plaque at the luminal interface eventually becomes sufficiently pronounced to disrupt blood flow, with consequences on nanoparticle targeting and cell state. This process is self-reinforcing as non-physiological shear stress patterns are known to promote atherogenesis, disease progression [121], and lead to leucocyte attachment and recruitment [122]. To study the influence of these hemodynamic conditions on the interaction of SMCs and ECs that can be found in close proximity presented in advanced lesions (due to SMC migration into the plaque) [123], Chen et al. placed a SMC-loaded collagen hydrogel co-seeded with ECs and exposed it to a vertical-step flow (VSF) chamber [124]. Due to the geometry and a specific flow rate, they were able to expose the matrix to a flow with the Reynolds number of 100, leading to a laminar flow recirculation without turbulence and resulting in shear stresses between 0, 1, and 7 dyne/cm^2^. In their set-up, they could detect that applying varying shear stress to co-cultured SMCs and ECs leads to an induction of adhesion molecules expression followed by an increased adhesion and transmigration of neutrophiles, lymphocytes, and monocytes under VSF [124].

With this VSF chamber, the behavior of nanoparticles in pathologically disturbed flow conditions could be monitored and the effect on particle approximation and adhesion due to changing flow conditions could be analyzed.

## 5. Tissue Engineering for Drug-Eluting Angioplasty Devices

**Figure 4:**
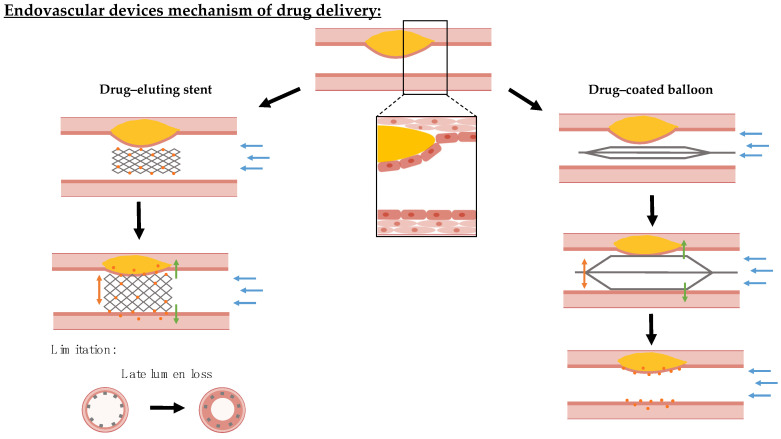
Overview mechanism of action of endovascular device drug delivery in stenosed blood vessels. Endovascular devices are coated with cytostatic drug coating that is locally delivered. Drug-eluting stents are permanently implanted at the target lesion and can slowly release their drug load from rate-limiting coatings. The implantation of stents is associated with safety concerns, since these can injure the endothelium leading to restenosis and malapposition increases the risk of thrombosis. In contrast, drug-coated balloons transfer their drug coating during a short inflation period and rely on slow coating dissolution to sustain local drug dosing within the tissue. For studying and assessing both devices, the biological–biochemical (green arrows) environment for drug effect, hemodynamic–microstructural (blue arrows) conditions for retention versus wash-out, and mechanical–geometrical properties (orange arrows) for expansion/inflation efficiency need to be mimicked by a tissue-engineered model.

Endovascular angioplasty balloons and stents offer an attractive means of propping open stenosed arteries and re-establishing physiological blood flow. To counteract the inflammatory and hyperplastic responses elicited by these mechanical procedures, local drug delivery has been added to these devices [125,126]. Despite stents and balloons being coated with the same range of anti-proliferative and anti-inflammatory drugs, the coating technologies and mechanism of drug delivery are divergent. While drug-eluting stents (DES) are implanted into the diseased vessel area and are able to release the drug slowly over time [127], drug-coated balloons (DCB) are inflated at the affected area for a short time of 1–3 min and need to transfer a sufficient amount of the drug during this time period [128], while, subsequently, providing mechanisms for its slow release within the target tissue [129,130]. This creates different advantages, risks, and control variables for optimization (Figure 4).

### 5.1. Model Attributes

Endovascular drug delivery depends on multiple factors like lesion composition, surface area, contact pressure, preoperative lesion treatment, and many more [131].

A suitable tissue-engineered disease model for testing drug-eluting devices should include not only the diseased biological–biochemical environment, but has to share the hemodynamic and geometrical features of the vascular system. The exposure to blood flow influences the balance of drug retention and washout [132,133], and the geometrical macrostructure of the surrounding vascular system influences device tracking and deployment at the target lesion [134]. These factors are of prime importance as regions with branches and bends are generally prone to develop atherosclerosis initially [135] and are, therefore, common sites for angioplasty.

Also, the mechanical properties and stiffness of the target lesion area itself determine the degree of device apposition to the vessel wall, and, thereby, the efficiency of drug delivery. For instance, very stiff lesions like calcified lesions withhold a greater risk of under-expansion of drug-eluting stents [136,137].

For drug-coated balloons, the coating transfer efficiency is presumed to be influenced by the tissue mechanics due to influencing the contact pressure between the device and the arterial wall [138]. In silico investigations show impact of vessel stiffness on contact pressure, proposing an impact on drug delivery [139].

The mechanical properties of healthy vessels are mainly dependent on the loading mechanics of its elastic fibers [140] and are considered to be in between a viscous liquid and an elastic solid [141].

With disease-caused alteration in the cellular and structural composition of the vessel wall, changes in the mechanical properties are also observed in atherosclerosis; especially, an increase in arterial stiffness is associated with a higher prevalence of cardiovascular disease [142]. Kobielarz et al. [143] analyzed the mechanical properties like failure stretch, stress, and stiffness of different human plaque and found that calcified plaques show highest stiffness followed by fibrotic plaques, while lipid plaques are the weakest and most stretchable plaques [143].

### 5.2. Available 2D and Animal Models

Due to their complex mechanism of drug delivery, most simple 2D cell cultures are not suitable for testing drug-delivery devices. Currently, simple tests of devices are performed on either ex vivo arteries [144] or cell-free hydrogels [145].

For more elaborate assessment and analysis of these devices, mainly animal models are used. Rodents like mice, rats, and rabbits are widely used to study devices for coronary applications due to their simple and affordable handling [146]. But due to the small dimensions of their vascular systems, these models are not suitable to test larger drug-eluting devices for peripheral arteries.

Porcine models provide a suitable geometry of the vascular structure and develop initial lesions or restenosis due to injury or hypercholesterolemic diet or a combination of both [147,148,149,150].

Despite showing similarities with early human atherosclerosis and in-stent restenosis, these animal models show deviations from human pathogenesis including disease progression or vascular response and are lacking capability to mimic advanced plaques [151].

### 5.3. Tissue-Engineered Models for Atherogenesis

Despite the focus of the current endovascular drug-delivery devices on the treatment of advanced atherosclerosis, their animal evaluation mostly relies on healthy models, with similar anatomical features. It is, therefore, instructive to examine here which already existing tissue-engineered models of early atherosclerosis disease have the potential to be utilized as test systems for these.

For efficient drug delivery, the mechanical properties and geometrical configuration of the global vascular system is determined [138]. Branching, bendings, and stenosis are influencing factors for device tracking and local drug delivery, while sufficient model diameter is necessary to test devices designed for the human vascular system.

One of the few tissue-engineered models that include branching are the branched TEBVs (brTEBVs) published by Lee et al. These brTEBVs were constructed from a base of iSMC mixed with collagen I and co-seeded with ECs, derived from human umbilical cord blood. Human endothelial progenitor cells (EPCs) were used to be converted into iSMCs by the expression of MYOCD [95].

To study the onset and simulate early stages of atherosclerosis, the engineered healthy tissue was exposed to enzyme-modified low-density lipoprotein (eLDL) and TNF-*α* to induce endothelial dysfunction, and trigger adhesion and transmigration of monocytes [152].

Applying pulsatile flow to the system, it could be demonstrated that the adhesion behavior of monocytes is greater at a higher angle of branching (45° vs. 60° and 80°). Being able to analyze the adhesion behavior of cells, this model could be utilized to not only perform drug screening but to also study nanoparticle behavior.

To date, brTEBV still have several shortcomings to solve before being utilized as models for evaluating angioplasty devices. In particular, their small diameter and lack of account for mechanical properties of the surrounding tissue limit the apposition of these devices. Nevertheless, this model holds the potential for further development into an important model for drug-eluting device testing.

One of the most sophisticated atherosclerotic in vitro models was published by Cho et al. [153] and consists of a complex three-layered vascular construct built from a monolayer of confluent endothelium and condensed SMC layer on a vascular tissue-derived decellularized extracellular matrix (VdECM). Due to their special in-bath coaxial bioprinting technique, they were able to form constructs with tunable geometry, including varying diameter, and the addition of stenosis or tortuous structures. Additionally, the model has the ability to apply varying shear stresses through different flow conditions [154].

This model enabled them to study the influence of factors like hemodynamic flow, inflammation markers (TNF-α), and hyperlipidemia (h-LDL) on endothelial dysfunction and to recapitulate the cellular and biochemical environment of early atherosclerosis. Triggering pathological signs of early atherosclerosis, the model was also utilized to evaluate the effect of cholesterol-lowering drugs on the pathogenesis and perform mechanistic studies [154]. Covering biological–biochemical, hemodynamic–microstructural, and mechanical–geometrical attributes of the physiological environment, this model contains the necessary complexity needed to not only test drugs and nanoparticles, but also drug-eluting devices.

### 5.4. Tissue-Engineered Models for Advanced Atherosclerosis

As described earlier, tissue-engineered models that could be implemented for endovascular drug-delivery device testing also need to mimic the mechanical and geometrical properties of the advanced lesion and the surrounding vascular system.

3D bioprinting techniques hold great potential for fabricating constructs that can mimic the geometric and mechanical properties of advanced diseased vessels. Patient-specific imaging data obtained from CT or MRI scans can be used to print a 3D phantom of the diseased vessel. Various groups used this approach to mimic stenosed coronary or carotid arteries and used them to validate their computational fluid dynamic models [155,156].

Guarnera et al. [157] published a novel approach using 5 different so-called *digital materials* (a mixture of soft and more stiff polymers that can be printed with a PolyJet printer) to print a phantom that mimics the mechanical properties of 5 components of the atherosclerotic plaque (adventitia, calcification, fibrous cap, fibrotic media, media, and lipid pool). They used this complex phantom to validate their numerical models of plaque mechanics.

These advances in the 3D bioprinting field are promising, but the current models lack in integration of biological component that is necessary for all types of treatment testing. Wu et al. [158] refined the formulation of a glycidyl methacrylated poly(vinyl alcohol) (PVAGMA) hydrogel mixed with cellulose nanocrystals (CNC) to generate a bioprintable ink that is tunable in tensile modules and shows no cytotoxic effect in vitro. Their arterial phantom showed stability and durability under cyclic loading with physiological flow conditions. Additionally, they generated phantoms with soft, hard, and mixed soft and hard segments, mimicking the mechanical properties of a diseased vessel. The acoustic properties of their material allowed for the in situ determination of local material stiffnesses in the phantoms using ultrasound.

All of the above-mentioned characteristics make the PVAGMA/CNC hydrogel the optimal bioprinting material that could be utilized to print mechanical and hemodynamic replicates of atherosclerotic disease models but have so far not included the biological–biochemical attributes that would render them applicable for testing of endovascular therapies.

As an approach to mimic the biological–biochemical environment of advanced plaques, Garcia-Sabaté et al. [159] used a 3D collagen matrix model with different collagen densities to study the role of macrophages and monocytes and their uptake of oxLDL taking the arterial stiffness in more advanced pathologies into account.

This was achieved by seeding human monocytic THP-1 cells in matrices with high (3 mg/mL) and low (1 mg/mL) collagen content representing stiffness of early and advanced lesions and determining the mechanical properties in cell-free collagen matrices. Cells were further differentiated to pro- (M1) and anti- (M2) inflammatory phenotypes and exposed to oxLDL. The oxLDL uptake, expression of relevant receptors, and secretion of inflammatory cytokines were analyzed. In the study, the authors found differences in cytokine expression of M1 macrophages, M2 macrophages, and THP-1 monocytes, as well as oxLDL uptake and lipid droplet size within macrophages in a matrix-dependent manner [159].

A further effort to generate advanced heterogenic plaque pathology (fibrotic plaque with lipid core) with tunable mechanical properties due to controlled collagen composition was published 2022 by Wissing et al. [160].

To create the model, human-derived vena spahena cells (HVSC) were seeded in a fibrin gel and cultured for 7 days under static conditions. After 7 days, a 2 mm diameter hole was punched out of the center of the sample and filled with fibrin to mimic the presence of a lipid core. Afterwards, the samples underwent different dynamic cultivation protocols, being exposed to static conditions, or intermittent or continuous conditions of 4% strain. Subsequently, the tissue content, collagen architecture, and mechanical properties were analyzed and showed physiological strain stiffening response and stiffness values. Most importantly, they established a protocol to modulate the collagen composition and organization in a controlled manner which can be used for mechanical testing or testing drug-eluting device coating transfer.

The notable ability of these sophisticated bioengineered models to mimic the mechanical properties of advanced atherosclerosis is an important step. However, in order for these 3D plaques to provide a model testing nanoparticles or drug-eluting devices they need to be integrated into a vascular-like structure that is exposable to physiological flow conditions.

## 6. Conclusions

In the testing and optimization of atherosclerotic treatment approaches, there is a growing demand for complex models that cannot sufficiently be filled by 2D cultures or animal models. Fortunately, tissue engineering can be harnessed to fill in this gap with stem cell-derived cell types providing a potentially limitless supply of materials for these models.

In this review, we illuminated the different attributes which a tissue-engineered disease model needs to incorporate in order to be adequate for atherosclerosis treatment development. Reviewing the different models that are available to date, it is apparent that there are already various sophisticated models which mimic the biological–biochemical reality and are, therefore, sufficient for screening and developing drug treatments, especially in atherogenesis (Table 4).

Available models of advanced atherosclerotic lesions are much more scarce and are also mostly designed to mimic only the biological–biochemical attributes of diseased tissue (Table 5). These models could be utilized for testing drugs but lack complexity which is needed to optimize nanoparticles and, especially, drug-eluting devices.

Most models described here only focus on simulating healthy or diseased vessels in one or two attributes. Only the model of Goa et al. [154] aims to simulate healthy vessels in all three attributes. If this model would be further developed on the biological–biochemical level to simulate diseased vessels, e.g., by incorporating the approached of Mallone et al. [102] for generating an advanced fibroatheroma or from Garcia-Sabaté et al. [159] to replicate the complex biochemical microenvironment of advance atherosclerotic plaques, it could be utilized for testing and optimizing drug-eluting devices.

We venture to predict that these models, combined with the different tremendous efforts that have been made in the disease-inspired vascular tissue engineering field within the last few years, will soon provide an important complement to 2D cell cultures and animal models.

## Figures and Tables

**Table 1:** Overview of recent clinical trials implementing TEBV [18,19,20,21,22,23,24,25,26].

Construct	Study Objective	Duration	Enrolled Patients	Reference
**Cavopulmonary shunts for congenital heart defect**				
Biodegradable scaffolds seeded with bone marrow cells	- Graft failure requiring intervention - Graft growth	24 month	42 patients	[18,19]
Biodegradable scaffolds seeded with bone marrow cells	- Safety and tolerability - Efficacy	12 month	25 patients	[20]
Biodegradable scaffold based vascular graft	- Graft failure requiring intervention - Graft growth	36 month	4 patients	[21]
**Arteriovenous shunts for end-stage renal disease**				
Patient derived cells in cell sheet-based tissue engineering method	- Safety assessment - Efficacy assessment	6 month	10 patients	[22]
Decellularized TEBV generated by culturing SCM onto a biodegradable scaffold	- Safety and tolerability - Patency rate	16 month	60 patients	[23,24]
Scaffold-free TEVG using sheets of extracellular matrix	- Safety assessment - Efficacy assessment	12–38 month	10 patients	[25]
**Vascular replacement for trauma damage**				
Human acellular vessels	- Primary patency - Frequency and severity of advice effects	36 month	72 patients	[26]

**Table 2:** Categorization of atherogenesis and advanced atherosclerosis.

	Research Use	Representation
**Atherogenesis**	Study on-set and initial progression of the disease	- Healthy arterial environment - Only native tissue cells present - Organized vascular structure (Figure 1A)
**Advanced atherosclerosis**	Optimize treatment of disease with severe clinical symptoms	- Stenosis (Figure 1B) - Migration of immune cells - Severe disorganization in vessel wall structure (Figure 1C) - Lipid pools, cholesterol crystals and necrotic core can be present (lipid plaque) - Fibrous cap/ calcification

**Table 3:** Requirements for each attribute and minimal set of attributes for testing each treatment approach.

Tissue Attribute	Model Requirements	Minimal Attribute for Testing		
		Drug	Nanoparticle	Drug-Eluting Device
Biological Biochemical	- Multicellular environment (SMC, endothelial cells, macrophages etc.) - Biochemical matrix - Addition of biochemical molecules (TNF-a, oxLDL, etc.)	√	√	√
Hemodynamic Microstructural	- Physiological flow conditions - Pathological inclusions - Flow-disrupting plaque - Lesion structure	X	√	√
Mechanical Geometrical	- Pathological mechanical properties (stress and strain modules etc.) - Complex vascular structure with insertion of branches, bendings and stenosis	X	X	√

**Table 4:** Summary of available tissue engineering models for studying atherogenesis and their applicability to be utilized for testing of drugs, nanoparticles, and drug-eluting devices.

Model for Atherogenesis	Application Area			Perspective
	Drugs	Nanoparticles	Drug-Eluting Device	
Cell cultures of iPSC derived from atherosclerotic patients [98,99,100]	√	X	X	-Biological
Co-cultured wall on a chip model with addition of IL-1b, TNF-a, oxLDL) [101]	√	X	X	-Biological–Biochemical
2–layered TEBV with TNF-a administration [117]	√	√	X	-Biological–Biochemical -Hemodynamic–Microstructural
2–layered scaffold based vascular graft, exposed to flow and TNF-a, LDL and HDL [118]	√	√	X	-Biological–Biochemical -Hemodynamic–Microstructural
2–layered tubular wall on a chip model with flow+ addition of LDL and macrophages [119]	√	√	X	-Biological–Biochemical -Hemodynamic–Microstructural
3–layered TEBV with addition of eLDL and TNF-a [120]	√	√	X	-Biological–Biochemical -Hemodynamic–Microstructural
2–layered TEBV with various geometries + addition of TNF-a/eLDL + flow [152]	√	√	/	-Biological–Biochemical -Hemodynamic–Microstructural -Macrostructural
3–layered coaxial in-bath bioprinting approach with tunable geometry + flow [154]	√	√	√	-Biological–Biochemical -Hemodynamic–Microstructural -Mechanical–Macrostructural

**Table 5:** Summary of available tissue engineering models for studying advanced atherosclerosis and their applicability to be utilized for testing of drugs, nanoparticles, and drug-eluting devices.

Model for Advanced Atherosclerosis	Application Area			Perspective
	Drugs	Nanoparticles	Drug-Eluting Devices	
3D bioprinting of hydrogels with tunable mechanical properties [157,158]	X	/	/	-Hemodynamic–Microstructural -Mechanical–Macrostructural
Hanging drop model for 3D fibroatheroma [119]	√	X	X	-Biological–Biochemical
Leucocyte recruitment monitoring in with 2-layered TEBV with exposure to WBC and TNA-a [103]	√	X	X	-Biological–Biochemical -Microstructural
Collagen hydrogel with co-cultured SCM/ECs under flow conditions [124]	√	√	X	-Biological–Biochemical -Hemodynamic
3D macrophages loaded collagen matrix models with tunable collagen densities exposed to oxLDL [159]	√	X	/	-Biological–Biochemical -Mechanical–Macrostructural
Mimicking mechanical properties of advanced fibrous cap model with myofibroblast loaded fibrin-collagen matrix [160]	√	X	/	-Biological–Biochemical -Mechanical–Macrostructural

## Data Availability

Data is contained within the review.
